# Sexual Orientation and Internalized Homophobia of Middle Aged and Older Gay and Lesbian Adults: The Role of Social Relationships

**DOI:** 10.1093/geronb/gbaf048

**Published:** 2025-03-07

**Authors:** Ella Cohn-Schwartz, Sigal Gooldin, Lian Meiry, Yaacov G Bachner

**Affiliations:** Gerontology Program, Department of Epidemiology, Biostatistics, and Community Health Sciences, School of Public Health, Ben-Gurion University of the Negev, Beer-Sheva, Israel; The Israeli Institute for Gender and LGBTQ Studies, Tel-Aviv, Israel; Gerontology Program, Department of Epidemiology, Biostatistics, and Community Health Sciences, School of Public Health, Ben-Gurion University of the Negev, Beer-Sheva, Israel; Department of Physiotherapy, Ben-Gurion University of the Negev, Beer-Sheva, Israel; Gerontology Program, Department of Epidemiology, Biostatistics, and Community Health Sciences, School of Public Health, Ben-Gurion University of the Negev, Beer-Sheva, Israel; (Social Sciences Section)

**Keywords:** LGBTQ, Mental health, Social networks

## Abstract

**Objectives:**

Research is needed about the role of families in relation to issues faced by middle-aged and older gay and lesbian adults, such as internalized homophobia and families of choice. This study examines how families of choice and families of origin shape experiences of internalized homophobia in midlife and older gay and lesbian adults, a population uniquely affected by the cumulative effects of societal stigma over the life course.

**Methods:**

We sampled 409 adults aged 50+ (range: 50–85) who self-identify as lesbian women or gay men. They answered a questionnaire about families of choice, families of origin, and internalized homophobia. Mediation models examined the role of families in the association of gender and internalized homophobia.

**Results:**

Lesbian women reported lower internalized homophobia compared to gay men, and this was partially explained by their greater likelihood of citing spouses/partners and children as close others. Friends in one’s family of choice were not associated with gender or internalized homophobia. Men were more likely to cite close siblings, and this was related to lower internalized homophobia, although siblings did not mediate the association of gender and internalized homophobia.

**Discussion:**

Higher internalized homophobia of gay men in later life might be partially explained by being less likely to have a spouse/partner and children, reflecting cumulative effects of lifelong discrimination and stigma. These findings could foster better interventions aimed at specific needs of aging men and women from sexual minorities, considering their life course experiences and social resources.

The world population, including a diverse population of sexual minorities, is rapidly aging. Older lesbian, gay, bisexual, and transgender (LGBT) individuals represent an understudied group within the larger aging population, with specific mental health and social needs, compounded by structural inequities (Fredriksen-Goldsen et al., 2019). Estimates from U.S. population-based surveys indicate that LGBT adults comprise between 2% and 6% of the adult population in the United States (Gates, 2014). Internalized homophobia, or the internalization of societal stigma against sexual minorities, becomes particularly salient as individuals age. Over time, discriminatory experiences accumulate, especially for those who came of age during periods of significant societal stigma (Mohr & Kendra, 2011; Wight et al., 2012).

Social relationships are integral to the mental health of heterosexual and nonheterosexual older adults alike (Santini et al., 2015; Schwarzbach et al., 2014; Shenkman et al., 2023). However, social relationships can be particularly complex for nonheterosexual older adults, who may lack traditional family support due to estrangement from their family of origin (Ismail et al., 2019). Instead, families of choice—close relationships with friends, partners, and other nonbiological kin—play a crucial role in their social networks (Fish et al., 2024). However, the effects of the LGBT population’s social relationships in later life, such as their families of choice, are not yet well understood. Therefore, this study aims to explore how these social relationships can be related to internalized homophobia. We will adopt a gendered lens by to examine the differences between gay men and lesbian women in these processes.

## Internalized Homophobia

Internalized homophobia (also referred to as internalized homonegativity and internalized heterosexism) refers to shame and low self-worth that can be experienced by LGBT adults due to negative public attitudes against sexual minorities (Mohr & Kendra, 2011). The Health Equity Promotion Model (HEPM; Fredriksen-Goldsen et al., 2014) emphasizes the interplay between structural, social, and individual factors in shaping LGBT health outcomes. It highlights internalized homophobia as a key factor linking societal stigma to health and well-being disparities (Fredriksen-Goldsen et al., 2014). Minority Stress Theory (Meyer, 2003) similarly identifies internalized homophobia as a major stressor resulting from belonging to a stigmatized minority population. Indeed, middle-aged and older adults with higher internalized homophobia tend to have more mental health problems compared to those with lower internalized homophobia (Lee et al., 2019; Newcomb & Mustanski, 2010; Perry et al., 2023; Ribeiro-Gonçalves et al. 2025; Wight et al., 2012). Moreover, adults in later life are particularly vulnerable to the negative effects of internalized homophobia, as there are indications that internalized homophobia is more strongly linked to mental health among older adults than younger adults (Lee et al., 2019; Newcomb & Mustanski, 2010). Possibly, these adults may have experienced a lifetime of discrimination, which can have cumulative negative effects of internalized homophobia (Newcomb & Mustanski, 2010). A cohort effect could also be at play, since adults from the baby boom and Generation X cohorts grew up in unique historical and social contexts when homosexuality was less acceptable and faced more discrimination, stigma, and the need to conceal their identity, further increasing the negative effects of internalized homophobia (Newcomb & Mustanski, 2010). The impact of internalized homophobia on midlife and older gay men and lesbian women may differ due to various intersecting factors. Intersectionality theory suggests that multiple social identities, such as sexual orientation, gender, and age, interact to create unique experiences of privilege or oppression (Bowleg, 2012). Recent studies have highlighted the importance of considering these intersecting identities when examining aging individuals from sexual minorities (Barbee et al., 2022).

## Social Relationships of Adults From Sexual Minorities

Adults from sexual minorities often have social relationships that differ from those of heterosexual adults. One aspect in need of more rigorous investigation is the family. Gay and lesbian adults often have limited ties with their families of origin due to rejection of their sexual identity or ambivalent ties, and many do not have children of their own (Fish et al., 2024; Ismail et al., 2019). With more limited ties, many gay and lesbian adults forge families of choice with unrelated others that either supplement or add to the relationships with family of origin (Fish et al., 2024; Gabrielson, 2011; Waldmann et al., 2011). “Families of choice” generally refers to social ties between friends and partners, in place of family of origin members. Although there is no one official definition of the concept, we will utilize the definition used in the MetLife study of LGBT Baby Boomers, as a group of people to whom one is emotionally close and consider “family” even though they are not biologically or legally related (Metlife, 2010).

Although important in the literature on sexual minorities, families of choice are not distinguished from other friends or explicitly defined in most quantitative research (Grossman et al., 2000; Ismail et al., 2019). Their centrality was illustrated by the finding that 64% of LGBT adults aged 45–64 had a “chosen family” (Metlife, 2010). Thus, families of choice warrant focused research attention to achieve a more rightful representation of these families, examining them in their own terms (Fish et al., 2024).

## Social Relationships and Internalized Homophobia

The HEPM underscores the protective role of meaningful social relationships as promoting resilience (Fredriksen-Goldsen & Kim, 2017; Fredriksen-Goldsen et al., 2014). Social relationships may also foster lower internalized homophobia, although current evidence is mixed. Previous research indicates that lower internalized homophobia among middle-aged and older gay men was related to having more tangible social support (Lyons & Pepping, 2017) and to having a partner (Wight et al., 2012). There are also indications that lower internalized homophobia is related to a better relationship quality and relationship satisfaction in same-sex romantic relationships (Cao et al., 2017; Li & Samp, 2019). However, support from family and friends was not found to be related to internalized homonegativity among gay men (Lyons & Pepping, 2017). Nevertheless, these studies mostly focused on men and did not explore specific members of one’s family of choice or family of origin. Close relationships, especially with chosen family who are friends and spouses/partners, and close family of origin members, could make one feel that their sexual orientation is accepted and help internalize this acceptance. By examining how families of choice and families of origin are differentially related to internalized homophobia, this study extends the HEPM to capture the unique social dynamics of adults from sexual minorities, such as their families of choice.

## Differences Between Gender Groups

We adopt a gendered lens to explore disparities between midlife and older gay cis-gender men and lesbian women. This is due to the complex nature of intersecting social positions such as sexual and gender identities in relation to social ties and health (Fredriksen-Goldsen et al., 2014). For example, older gay men were found to report more loneliness than did older lesbian women, although the reverse is usually found in the general population (Shnoor & Berg-Warman, 2019). The gender difference between gay men and women could increase in later life due to the greater effects of HIV/AIDS on the social relationships of gay men than on lesbian women (Tester, 2018), as well as growing up in a period when there were greater barriers for gay men to have children (Barbee et al., 2022; Shenkman et al., 2023). An examination of both gender groups is additionally imperative in light of the discrepancies in research attention, with most studies on internalized homophobia among middle-aged and older adults focusing on men (e.g., Lyons & Pepping, 2017; Perry et al., 2023; Ribeiro-Gonçalves et al., 2019; Wight et al., 2012), although one study that examined both gender groups indicated that men report higher internalized homophobia compared to women (D’Augelli et al., 2001).

To sum, this study aims to explore the social relationships of middle-aged and older gay men and lesbian women and their associations with internalized homophobia. Specifically, we will examine how social relationships might mediate gender differences in internalized homophobia. We hypothesize that: (a) Lesbian women will report lower internalized homophobia compared to gay men. (b) Lesbian women will report more social resources compared to gay men. (c) Aging adults with more social resources will report less internalized homophobia. (d) The lower internalized homophobia among lesbian women will be partly explained by their greater social resources.

## Method

### Participants and Procedure

The study was conducted in Israel. We collected the data via an online questionnaire to maintain the anonymity of the participants. To reach members of the gay and lesbian community, the study was published on social media, through WhatsApp groups of the LGBT community, Facebook groups, and paid online advertising. The respondents were also asked to send the survey link to other potential respondents in their social circle using the “snowball” method. Participants were recruited through collaboration with the Israeli Institute for Gender and LGBTQ Studies. Respondents who answered the survey had the option to leave their contact information and receive a gift voucher worth NIS 85 (~23$). Data collection received approval from the institutional review board of Ben-Gurion University. Participants signed an informed consent form at the beginning of the questionnaire, in which they were assured that their identities would be safeguarded and that the gathered information would be used for research purposes only. Most of the questionnaires were completed in Hebrew. The current study is part of a larger study, which included an online survey that was made up of mostly close-ended questionnaires about the social relationships of LGBT adults aged 50+. The data was collected between January and August 2023. The inclusion criteria were: age 50 or above, ability to read and understand one of the survey languages (Hebrew, English, or Arabic), and self-identification as gay man or lesbian women. Exclusion criteria were: under the age of 50 and self-identification other than a gay man or a lesbian women.

A total of 498 gay men and lesbian women began the survey. Of these, 411 completed over 90% of the survey. The 90% criterion was set because it indicated that participants reached the sociodemographic questions at the end of the survey, without necessarily completing the final section where they provide their contact information to receive a gift voucher. Of these 411 people, 409 had complete information on the study variables. Thus, the analytical sample focused on 409 respondents. Missing data analysis indicated that those who completed over 90% of the survey were older compared to those who completed less than 90% of the survey (*p* < .05). They did not differ in terms of their gender or citing of children, siblings, partners of friends in their close networks.

### Measures

*Internalized homophobia* was measured using the sexual identity distress scale (Wright & Perry, 2006). This scale contains seven items, four targeting negative feelings toward being gay (e.g., “I wish I were not attracted to the same sex”) and three targeting positive feelings (e.g., “I have a positive attitude about my sexual orientation”), which were recoded such that a higher score indicated a more negative attitude. Response options ranged between (1) *strongly disagree* to (5) *strongly agree*. The scale was translated into Hebrew using back-translation. First, a behavioral scientist translated the scale from English to Hebrew. Then, the second translator independently back-translated it into English. The two translators discussed any differences between the versions and agreed on the most accurate and culturally appropriate phrasing. The scale was translated from English into Arabic by a professional translator. The responses to all items were averaged so that higher scores indicated greater internalized homophobia. The scale demonstrated high internal reliability (*α* = 0.84). In addition, factor analysis confirmed that the scale consists of a single factor.

#### Social relationships

We assessed participants’ social relationships by first asking whom they consider to be a close biological family\family of origin member. We created two dichotomous variables, which indicated whether a child or a sibling was mentioned, since these were the two most prevalent categories among family of origin members. We also asked participants about whom do they consider to be in their “family of choice,” defined as “people with whom you are emotionally close and consider ‘family’ though they are not biologically related to you” (Metlife, 2010). We created two dichotomous variables indicating a spouse/partner and a friend, which were the two most prevalent categories among family of choice members.

#### Gender

We asked participants to indicate their gender and focused on those who responded either man or woman. We also asked them to indicate their sexual orientation and focused on those who self-identified as gay or lesbian.

#### Covariates

We asked participants about their age (years, continuous variable), level of education (below high education\high education), and whether they have any medical chronic conditions (yes\no). Financial status was gleaned by asking how well their household can make ends meet. Response options ranged between (1) “Cannot make ends meet” and (4) “Excellent,” such that higher scores represent a better perceived financial status.

### Data Analysis

First, we employed descriptive statistics, followed by bivariate associations of the study variables with internalized homophobia and with gender. These associations were examined using Pearson, Spearman, or chi-square tests for continuous, ordinal, and categorical variables, respectively. We then examined the mediation models using mediation analyses as part of a path analysis method. We ran a structural equation model, in which gender predicted the citing of the different members in the family of origin and family of choice, and these in turn predicted internalized homophobia. We estimated the mediating effects of the social networks between gender and internalized homophobia. The model controlled for the mentioned covariates. We used the Lavaan package in R for structural equation model estimation (Rosseel, 2012). When binary endogenous variables are used in the model, Lavaan uses diagonally weighted least squares to estimate the model parameters, and the full weight matrix to compute robust standard errors, and a mean-and variance-adjusted test statistic. This method uses listwise deletion. Model fit was evaluated based on the criteria of CFI >0.95, SRMR <0.08, and RMSEA <0.08 (Hooper et al., 2008).

## Results

Table 1 presents descriptive information for the study variables and for participants’ sociodemographic characteristics. The participants reported relatively low internalized homophobia of 1.8 (range: 1–4). About two-thirds had a spouse/partner (62%) or a friend (65%) in their family of choice. A lower percentage of 53% cited a child in their close family of origin, while a higher share of 71% mentioned a sibling as a close family of origin member. The participants were composed of 44% women. They were aged 58 on average (range: 50–85), with a majority of 75% having higher education, being in a relatively good financial status (almost 3 of 4), and two-thirds reported having a chronic condition.

**Table 1.** Sample Characteristics of the Study and Bivariate Analyses of Their Associations With Internalized Homophobia (*N* = 409)

Variable	Mean (*SD*)	%	Range	Bivariate analyses internalized homophobia	
				*t*	*r*
Internalized homophobia	1.77 (0.69)		1–4		
Spouse/partner in family of choice		62.35		3.86***	
Friend in family of choice		65.28		–0.05	
Child in close family of origin		52.81		3.96***	
Sibling in close family of origin		70.90		1.18	
Gender (women)		43.52		4.24***	
Age	58.20 (6.84)		50–85		–0.04
Education (high education)		75.06		1.15	
Financial status	2.75 (0.86)		1–4		–0.03
Has chronic conditions		63.33		0.33	

*Note.* The bivariate associations with internalized homophobia were Pearson correlations (*r*) for continuous variables and *t* tests for binary variables (*t*); *p < .05. **p < .01. ***p < .001.

Table 1 also shows the bivariate associations of the study variables with the outcome variable of internalized homophobia. A significant association with having a spouse/partner in the family of choice indicates that those who had a spouse/partner also reported lower internalized homophobia, while the presence of a friend in one’s family of choice was not related to internalized homophobia. Citing a child as a close family of origin member was also related to internalized homophobia, such that those who mentioned a child had lower internalized homophobia, while a sibling was not related to internalized homophobia. Gender also emerged as a significant factor, with women reporting lower internalized homophobia compared to men (see also Table 2). The covariates were not significantly related to internalized homophobia.

**Table 2.** Gender Differences in the Study Variables of Internalized Homophobia, Social Ties and the Covariates

Variable	Men		Women		*t*	*χ* ^2^
	Mean (*SD*)	%	Mean (*SD*)	%		
Internalized homophobia	1.89 (0.74)		1.61 (0.58)		4.21***	
Spouse/partner in family of choice		54.74		71.51		11.36***
Friend in family of choice		65.95		64.25		0.06
Child in close family of origin		38.36		72.07		44.74***
Sibling in close family of origin		76.29		63.13		7.81**
Age	58.36 (6.71)		58.13 (7.10)		0.33	
Education (high education)		77.06		72.63		0.84
Financial status	2.78 (0.88)		2.71 (0.84)		0.77	
Has chronic conditions		62.50		64.80		0.14

*Note*: *SD* = standard deviation, *t*/*χ*^2^ = bivariate tests of gender differences: *t* tests for continuous variables and chi-square tests for binary variables; *p < .05. **p < .01. ***p < .001.

Next, Table 2 shows gender differences in the study variables. Women were more likely to have a spouse/partner in their family of choice than men, although no gender differences emerged in relation to having a friend in one’s family of choice. Women were almost twice more likely to have a child in their close family of origin, while men tended to a larger degree to mention a sibling in their close family of origin. There were no significant gender differences in the covariates—age, education, financial status, or having chronic conditions.

The final stage of analyses focused on path analyses models of the mediation mechanisms, as shown in Table 3. The first model examined the main effects in relation to internalized homophobia, without the mediation of social relationships. The model had excellent fit to the data (CFI = 1.00, RMSEA = 0.00, SRMR = 0.00). The results showed that gender had a direct association with internalized homophobia, such that women had lower internalized homophobia. Having a spouse/partner in the family of choice and having a child or a sibling as a close family of origin member, were related to lower internalized homophobia.

**Table 3.** Results From the Mediation Model of the Association of Internalized Homophobia With Gender and Mediation by Social Ties

Path	Model 1			Model 2			Model 3		
	*B* (*SE*)	CI	Beta	*B* (*SE*)	CI	Beta	*B* (*SE*)	CI	Beta
Gender (women) → Int. homophobia	–0.20 (0.07)	–0.34, –0.06	–0.14**	–0.16 (0.08)	–0.33, 0.00	–0.12*	–0.16 (0.09)	–0.33, 0.01	–0.12+
Spouse/partner family of choice → Int. homophobia	–0.22 (0.07)	–0.36, –0.08	–0.16**	–0.13 (0.05)	–0.22, –0.04	–0.20**	–0.15 (0.05)	–0.24, –0.05	–0.21**
Friend family of choice → Int. homophobia	–0.03 (0.07)	–0.17, 0.10	–0.02	–0.03 (0.05)	–0.12, 0.06	–0.04	–0.03 (0.05)	–0.13, 0.06	–0.05
Child close family of origin → Int. homophobia	–0.18 (0.07)	–0.32, –0.04	–0.13*	–0.10 (0.04)	–0.19, –0.02	–0.16*	–0.11 (0.05)	–0.21, –0.01	–0.16*
Sibling close family of origin → Int. homophobia	–0.15 (0.07)	–0.30, –0.01	–0.10*	–0.10 (0.04)	–0.18, –0.01	–0.14*	–0.11 (0.05)	–0.19, –0.02	–0.15*
Gender (women)									
** →** Spouse/partner family of choice				0.46 (0.13)	0.20, 0.71	0.22***	0.47 (0.12)	0.24, 0.70	0.23***
** →** Friend family of choice				–0.06 (0.13)	–0.31, 0.19	–0.03	–0.06 (0.13)	–0.31, 0.19	–0.03
** →** Child close family of origin				0.88 (0.13)	0.63, 1.14	0.40***	0.83 (0.10)	0.64, 1.02	0.41***
** →** Sibling close family of origin				–0.38 (0.13)	–0.64, –0.12	–0.19**	–0.37 (0.13)	–0.61, –0.12	–0.18**
R^2^	0.09			0.12			0.13		

*Note*: *B* = coefficient; *SE* = standard error; Beta = standardized coefficient; CI = confidence intervals; Int. Homophobia = internalized homophobia; Model 2 added the mediation effects; Model 3 added the covariates of age, education, financial status, and chronic conditions; +*p* < .10. *p < .05. **p < .01. ***p < .001.

The second model examined mediation without the covariates. The model had excellent fit to the data (CFI = 1.00, RMSEA = 0.00, SRMR = 0.00). The results showed that when taking into consideration the mediating associations, gender had a nearly significant direct association with internalized homophobia (*p* = .055). In relation to gender differences in social ties, women were more likely to mention a spouse/partner in the family of choice and a child in their close family of origin, while they were less likely to indicate a sibling as a close family of origin member. In turn, having a spouse/partner in the family of choice was related to lower internalized homophobia. Having a close child or a sibling was also related to lower internalized homophobia.

The indirect effect of having a spouse/partner in one’s family of choice was significant, such that mentioning a spouse/partner explained some of the association of gender with internalized homophobia (*B*(*SE*) = –0.058 (0.026), *p* = .027). The indirect effect of having a close child was also significant, and it explained some of the association of gender with internalized homophobia (*B*(*SE*) = –0.094 (0.041), *p* = .022). The indirect effect of citing a sibling was not significant (*B*(*SE*) = 0.034 (0.020), *p* = .086).

**Figure 1.**
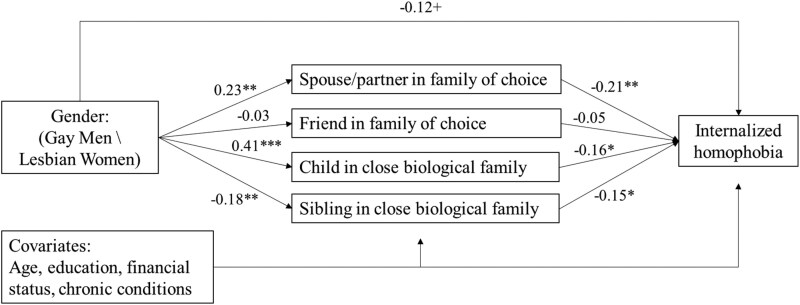
Model of the study results of the association of internalized homophobia with gender and mediation by social ties. *Note.* The numbers indicate standardized coefficients; +*p* < .10, *p < .05, **p < .01, ***p < .001.

These results were also seen in the third model, which added the covariates. This model also showed excellent fit to the data (CFI = 1.00, RMSEA = 0.00, SRMR = 0.00). Gender had a nearly significant direct association with internalized homophobia (*p *= .060). Mentioning a spouse/partner in one’s family of choice mediated the association of being a woman with lower internalized homophobia (*B*(*SE*) = –0.068 (0.028), *p* = .015), as did citing a close child (*B*(*SE*) = –0.093 (0.043), *p* = .032). The main study results are also graphically presented in Figure 1.

## Discussion

The current study set out to examine the complex relationship of gender, social relationships, and internalized homophobia among middle-aged and older lesbian and gay adults. The main findings indicated that lesbian women reported lower internalized homophobia, and this was partially explained by their greater likelihood of having close relationships with their spouse/partner and children. Friendship ties were not associated with gender or internalized homophobia, while the relationship with siblings showed a different trend—men were more likely to cite siblings as close, and citing siblings was related to lower internalized homophobia. Siblings, however, did not mediate the association of gender and internalized homophobia.

In accordance with the first study hypothesis, gay men had higher internalized homophobia compared to lesbian women. Although a previous study also found indications that men report higher internalized homophobia compared to women (D’Augelli et al., 2001), its findings were based on data collected from adults who were aged 60+ in 1997–8, having been born in much earlier periods in terms of gay rights, prior to World War 2. The current sample belongs to the baby boom generation and even generation X. They belong to the generation that experienced dramatic changes in gay life and matured as gay/lesbian adults into a context that was more accepting and liberal. The HEPM suggests that historical context plays a significant role in shaping health outcomes; thus, understanding these generational differences is essential for contextualizing our findings (Bowleg, 2012). Other studies of internalized homophobia among middle-aged and older adults mostly focus on men and lack a gender comparison (e.g., Perry et al., 2023; Ribeiro-Gonçalves et al., 2019). Thus, our investigation shows that even among adults in the baby boom cohort, lesbian women feel less internalized homophobia. One possible explanation is that men experience more sexual orientation-based victimization than women and consequently might internalize societal antihomosexual attitudes (Newcomb & Mustanski, 2010). The current study, however, offers an additional explanation which focuses on the different social resources of men and women.

### Gender Differences in Social Ties and Internalized Homophobia

The study findings also indicated gender differences in relation to social ties. Women were more likely to have a close spouse/partner and children, while men were more likely to cite a close sibling, and no gender differences were found in relation to friends in the family of choice. These results corroborate our second hypothesis since women reported more close social resources in the shape of a spouse/partner and child, which are often the main sources of support available as individuals grow old (Fischer & Beresford, 2015). However, unlike our hypothesis, gay men might partly make up for this shortcoming by having a close relationship with their siblings and friends. Nevertheless, these findings can attest to the increased risk of social isolation of gay men and strengthen the emphasis of the HEPM on the importance of considering intersecting social positions such as gender and sexual orientation (Fredriksen-Goldsen et al., 2014).

Age should also be added as an additional intersecting stressor. Belonging to an older cohort has likely also placed these adults, especially the men, at a greater risk of isolation due to the accumulation of discrimination experiences, growing in a period of more social stigma and barriers, as well as the challenges of aging such as retirement, failing health and increased need of medical services (Bowleg, 2012; Wight et al., 2012). It is noteworthy that these findings contrast with what is often seen among heterosexual aging adults, where men are more likely to have a close spouse/partner and women are more likely to have close friends (Cohn-Schwartz & Naegele, 2023; Fischer & Beresford, 2015). Thus, these trends indicate the different social situations of gay and lesbian adults as they grow older.

In addition, the current study is innovative in its in-depth examination of the mechanisms underlying the associations of gender and internalized homophobia. The results show that in accordance with our third hypothesis, having more social resources—namely a close spouse/partner, child, and sibling—was related to lower internalized homophobia. In accordance with our fourth hypothesis, this link can partially explain the gender differences in internalized homophobia—some of the lower internalized homophobia of lesbian women was explained by having a close relationship with a spouse/partner and children. These findings align with the HEPM which posits that strong social ties can foster resilience and mitigate internalized stigma (Fredriksen-Goldsen et al., 2014). Our study emphasizes the supportive roles of spouses/partners and children in reducing feelings of internalized homophobia. From the perspective of attachment theory (Mikulincer & Shaver, 2009), support from attachment figures can send the message that the world is safe and that others are available and can be trusted. A spouse/partner and children might be close and long-lasting ties whose acceptance is central to feeling that one’s sexual orientation is accepted. This is particularly important for aging adults, given their increased likelihood of experiencing deteriorating health and increased reliance on others.

This study was conducted in Israel, which is considered a familistic and pronatalist society that prioritizes traditional family structures (Lavee & Katz, 2003). Despite general support for families, older gay men are less likely to be in a relationship or to be parents, possibly due to gay men being specifically targeted by discriminatory practices in the field of adoption or surrogacy, limiting their access to fatherhood, while women can get state support for fertility treatments (Shenkman et al., 2023). Gay men also face greater discrimination due to widespread patriarchal and masculine norms in a more traditional society (Sion & Ben‐Ari, 2009). Thus, this cultural context could have further enhanced gender differences, while in societies with greater legal recognition of same-sex partnerships and parental rights, gay men could experience stronger social support networks and lower internalized homophobia. Furthermore, the familistic norms in the Israeli context may amplify the protective role of spouses/partners and children observed here. In individualistic cultures, characterized by a lower emphasis on familial ties and stronger LGBT community networks, friendships in families of choice could play a more central mediating role. Future cross-cultural research should explore how these dynamics manifest in different cultural contexts.

Contrary to some previous research and our expectations, friendship ties were not associated with internalized homophobia. However, these findings align with a previous finding that support from friends was not related to internalized homonegativity (Lyons et al., 2017). Possibly, friendship ties would more likely belong to the LGBT community and may not necessarily challenge internalized negative beliefs about one’s sexual orientation. The shared experiences within these friendships might even reinforce, rather than alleviate, internalized stigma. Ties with a spouse/partner, children, and siblings, on the other hand, could represent adhering to more heteronormative norms and relationship structures. Acceptance from these people could be particularly powerful in countering internalized homophobia, as they represent recognition from mainstream society. In addition, a spouse/partner and children could be more likely to provide tangible or practical support, which were previously found to be associated with lower internalized homonegativity. Friendships might not provide the same level of practical support (Lyons & Pepping, 2017).

The current study has several implications. Our findings could facilitate better-targeted interventions and policy aimed at specific needs of men and women from sexual minorities. Policy initiatives for alleviating social isolation could especially target gay men who don’t have a spouse/partner and children, since they might face greater risks of internalized homophobia (Rapid Response Service, 2020). Such initiatives could help by alleviating social isolation and improving sexual minorities individuals’ perceptions of their sexual orientation. The current findings also support the distinction of intervention objectives for gay men and women regarding social support. For example, since men emerged as having fewer close ties with a spouse/partner, while having closer ties with their siblings, interventions could focus more on helping gay men find a spouse/partner, while also emphasizing the importance of cultivating good relationships with one’s siblings. These goals could be achieved during cognitive-behavioral interventions tailored toward gay men, which promote emotional support from others, improving the relationship with existing ties and cultivating new ties (Flentje, 2020; Satterfield & Crabb, 2010; Yang et al., 2024).

### Study Limitations

This study has several limitations that should be noted. A primary challenge when conducting research with the LGBT community is the ability to obtain a sufficient sample size from a group that has been historically marginalized and might be reluctant to provide personal information for a survey (Fredriksen-Goldsen et al., 2019). Thus, our sample is probably not representative, as it is likely biased toward adults who are “out” and publicly acknowledge their sexual identity. Moreover, we collected data online, which allowed for anonymity of the participants and a wider reach geographically, but also likely limits the representativeness of our sample to adults who have better digital orientation. Similar to a previous study of LGBT adults in Israel (Shnoor & Berg-Warman, 2019), our sample has a relatively large share of adults who have high education and are in a good financial status. To cope with these inherent limitations, we used a snowballing method, in addition to actively reaching out through LGBT organizations. We also emphasized the confidentiality of the questionnaires and the importance of the research (Fredriksen-Goldsen et al., 2013; Shnoor & Berg-Warman, 2019).

An additional potential limitation is that the temporal causal process may differ from our hypothesis. For example, it is possible that individuals with lower internalized homophobia will be more likely to form close relationships, rather than the other way around (Perry et al., 2023). That is, their homophobia-influenced self-concept regarding their lovability and value could inhibit them from creating and maintaining meaningful connections (Satterfield & Crabb, 2010). Future research using longitudinal data should examine such processes of bidirectional temporal processes. In addition, we recognize the importance of considering racial and ethnic identities in our research. To address this, we distributed the questionnaire in Arabic, in addition to Hebrew and English, with the intention of reaching gay and lesbian Arab adults. Unfortunately, despite our efforts, we were unable to reach gay or lesbian adults from the Arab population. This is likely due to the more conservative nature of this community, where mid-life and older adults, in particular, are less likely to engage with LGBT organizations. Future research should explore additional strategies to better engage this population. We also note that there are potential unmeasured confounders that could influence both social relationships and internalized homophobia, such as prior experiences of discrimination, social stigma, or mental health status. Another limitation is that the framing of the measure on families of origin and chosen families is missing the legality aspect of these concepts.

We also note that we focus on cis-gender gay men and lesbian women as the study specifically examines the intersection of gender and sexual minority status. Including bisexual individuals would have introduced additional dimensions, such as identity fluidity and varying experiences of stigma, which extend beyond the scope of the current study, as well requiring a larger sample. Future studies should explore additional sexual and gender minority groups to provide a more comprehensive understanding of social relationships within the broader LGBTQ+ community.

### Summary

This study contributes to the currently limited understanding of the social ties and internalized homophobia among middle-aged and older gay and lesbian adults. The findings revealed that lesbian women experienced lower levels of internalized homophobia, partly due to stronger bonds with their spouse/partner and children. These findings contribute to the understanding of how social relationships shape the experiences of sexual minorities in midlife and later years, with implications for more personally tailored mental health interventions. Given the pain and suffering experienced by this understudied population, it is crucial to find ways to support aging gay and lesbian adults. This study, by identifying the relevant issues, provides a good place to start.

## Data Availability

This study is not preregistered. The data and materials are not available online because the authors have not completed their original work with the data set. We are happy to share the data upon request.
